# Combination laser interstitial thermal therapy plus stereotactic radiotherapy increases time to progression for biopsy-proven recurrent brain metastases

**DOI:** 10.1093/noajnl/vdac086

**Published:** 2022-06-02

**Authors:** Matthew M Grabowski, Ethan S Srinivasan, Eugene J Vaios, Eric W Sankey, Balint Otvos, Daria Krivosheya, Alex Scott, Michael Olufawo, Jun Ma, Elena I Fomchenko, James E Herndon, Albert H Kim, Veronica L Chiang, Clark C Chen, Eric C Leuthardt, Gene H Barnett, John P Kirkpatrick, Alireza M Mohammadi, Peter E Fecci

**Affiliations:** Department of Neurosurgery, Rose Ella Burkhardt Brain Tumor & Neuro-Oncology Center, Cleveland Clinic & Case Comprehensive Cancer Center, Cleveland, Ohio, USA; Department of Neurosurgery, Duke University Medical Center, Durham, North Carolina, USA; Department of Radiation Oncology, Duke University Medical Center, Durham, North Carolina, USA; Department of Neurosurgery, Duke University Medical Center, Durham, North Carolina, USA; Department of Neurosurgery, Rose Ella Burkhardt Brain Tumor & Neuro-Oncology Center, Cleveland Clinic & Case Comprehensive Cancer Center, Cleveland, Ohio, USA; Department of Neurosurgery, Rose Ella Burkhardt Brain Tumor & Neuro-Oncology Center, Cleveland Clinic & Case Comprehensive Cancer Center, Cleveland, Ohio, USA; Department of Neurosurgery, Washington University School of Medicine, St. Louis, Missouri, USA; Department of Neurosurgery, Washington University School of Medicine, St. Louis, Missouri, USA; Department of Neurosurgery, University of Minnesota, Minneapolis, Minnesota, USA; Department of Neurosurgery, Yale University School of Medicine, New Haven, Connecticut, USA; Department of Biostatistics and Bioinformatics, Duke University School of Medicine, Durham, North Carolina, USA; Department of Neurosurgery, Washington University School of Medicine, St. Louis, Missouri, USA; Department of Neurosurgery, Yale University School of Medicine, New Haven, Connecticut, USA; Department of Neurosurgery, University of Minnesota, Minneapolis, Minnesota, USA; Department of Neurosurgery, Washington University School of Medicine, St. Louis, Missouri, USA; Department of Neurosurgery, Rose Ella Burkhardt Brain Tumor & Neuro-Oncology Center, Cleveland Clinic & Case Comprehensive Cancer Center, Cleveland, Ohio, USA; Cleveland Clinic Lerner College of Medicine of Case Western Reserve University, Cleveland, Ohio, USA; Department of Radiation Oncology, Duke University Medical Center, Durham, North Carolina, USA; Duke Center for Brain and Spine Metastasis, Durham, North Carolina, USA; Department of Neurosurgery, Rose Ella Burkhardt Brain Tumor & Neuro-Oncology Center, Cleveland Clinic & Case Comprehensive Cancer Center, Cleveland, Ohio, USA; Cleveland Clinic Lerner College of Medicine of Case Western Reserve University, Cleveland, Ohio, USA; Department of Neurosurgery, Duke University Medical Center, Durham, North Carolina, USA; Duke Center for Brain and Spine Metastasis, Durham, North Carolina, USA

**Keywords:** brain metastasis, LITT, progression, stereotactic radiotherapy, tumor recurrence

## Abstract

**Background:**

Improved survival for patients with brain metastases has been accompanied by a rise in tumor recurrence after stereotactic radiotherapy (SRT). Laser interstitial thermal therapy (LITT) has emerged as an effective treatment for SRT failures as an alternative to open resection or repeat SRT. We aimed to evaluate the efficacy of LITT followed by SRT (LITT+SRT) in recurrent brain metastases.

**Methods:**

A multicenter, retrospective study was performed of patients who underwent treatment for biopsy-proven brain metastasis recurrence after SRT at an academic medical center. Patients were stratified by “planned LITT+SRT” versus “LITT alone” versus “repeat SRT alone.” Index lesion progression was determined by modified Response Assessment in Neuro-Oncology Brain Metastases (RANO-BM) criteria.

**Results:**

Fifty-five patients met inclusion criteria, with a median follow-up of 7.3 months (range: 1.0–30.5), age of 60 years (range: 37–86), Karnofsky Performance Status (KPS) of 80 (range: 60–100), and pre-LITT/biopsy contrast-enhancing volume of 5.7 cc (range: 0.7–19.4). Thirty-eight percent of patients underwent LITT+SRT, 45% LITT alone, and 16% SRT alone. Median time to index lesion progression (29.8, 7.5, and 3.7 months [*P* = .022]) was significantly improved with LITT+SRT. When controlling for age in a multivariate analysis, patients treated with LITT+SRT remained significantly less likely to have index lesion progression (*P* = .004).

**Conclusions:**

These data suggest that LITT+SRT is superior to LITT or repeat SRT alone for treatment of biopsy-proven brain metastasis recurrence after SRT failure. Prospective trials are warranted to validate the efficacy of using combination LITT+SRT for treatment of recurrent brain metastases.

**Key Points**

- LITT is a validated therapeutic option for metastatic brain tumor recurrence after prior SRT.
- LITT+SRT improves freedom from local progression for SRT-failed brain metastases.

**Importance of the Study**

The following study reports clinical outcomes of biopsy-proven recurrent brain metastases treated with repeat stereotactic radiotherapy (SRT), laser interstitial thermal therapy (LITT), or the combination of LITT+SRT. Prior literature investigating the efficacy of LITT+SRT has been limited to a single case series. Studies of repeat SRT alone have largely depended on less reliable imaging techniques for identification of recurrent tumor versus radiation necrosis, which limits their generalizability given potential heterogeneity in the cohort. With strict inclusion of only biopsy-proven recurrences across all treatment groups, this study uniquely and significantly demonstrates improved freedom from local progression in patients treated with the combination of LITT+SRT. This finding represents a novel and meaningful contribution to the literature on recurrent brain metastases. Future prospective work validating the efficacy of LITT+SRT would offer a promising development for a patient cohort with currently limited treatment options.

Brain metastases currently affect 10%–20% of solid tumor cancer patients and comprise more than 50% of all intracranial tumors in adults.^1^ Management of recurrent brain metastasis is now a substantial and growing neuro-oncologic challenge, with cases expected to rise as systemic therapy for extracranial disease extends survival.^2^ Yet, there remains a lack of data to guide treatment strategies in patients with recurrent brain metastasis previously managed with stereotactic radiotherapy (SRT). The integration of modern targeted therapy using SRT and laser interstitial thermal therapy (LITT) presents a potential solution to achieve durable local control in this growing patient population.

Standard of care for newly diagnosed brain metastases is dictated by tumor location and symptomatology, and typically includes a combination of radiotherapy ± surgery.^2,3^ Though brain metastases are historically considered chemotherapy-resistant, blood–brain barrier-permeable constructs, targeted therapies, and immunotherapies have yielded promising early results as clinical trials are ongoing.^2^ Whole brain radiotherapy (WBRT) was previously the standard of care for patients with multiple brain metastases. However, WBRT has increasingly been replaced by SRT due to its associated neurocognitive toxicity.^4^ By delivering ablative single or multifraction doses of radiation in a conformal manner to the target lesion or resection bed, SRT enables durable local control rates with reduced neurocognitive toxicity.^5–8^ While initially restricted to patients with limited brain metastases, accumulating evidence from randomized trials and technologic advances such as single-isocenter multitarget treatment have broadened the indications for SRT to patients with multiple brain metastases.^9,10^ However, despite these efforts, brain metastasis remains a morbid and fatal disease, with median survival less than 12 months. Additionally, recurrence at sites previously treated with SRT occurs in 14%–31% of patients.^7,9–11^ The effective management of recurrent disease, using repeat irradiation, craniotomy, or LITT, is an active area of investigation.^12–17^

LITT was initially developed for the treatment of deep-seated tumors, utilizing a laser catheter stereotactically placed under magnetic resonance imaging (MRI) guidance. Once within the lesion, the activated laser thermally ablates the tissue as MR thermometry is followed to monitor the temperature change in real-time.^18–22^ Since its early clinical translation, intracranial applications of LITT have expanded to include both metastatic and primary brain tumors, radiation necrosis (RN), and epilepsy.^12,23–27^ Along with its direct cytotoxic effect, the approach offers a minimally invasive alternative for patients who are otherwise not candidates for larger surgical approaches, and is associated with short hospital stays and low patient morbidity.^12,13,18,26–29^ Thus far, studies of LITT for metastatic tumor recurrence after SRT demonstrate positive results with equivalent local control and overall survival (OS) compared to craniotomy.^13,30–32^ Given these findings, we aimed to investigate whether integrating LITT with SRT improves outcomes for patients with recurrent metastatic tumors previously treated with radiation. We hypothesized that combined modality therapy would improve local control compared to using LITT or SRT alone in patients with recurrent brain tumors.

## Materials and Methods

### General Study and Patient Information

After IRB approval, a multi-institutional, retrospective cohort review was performed on patients who underwent LITT+SRT, LITT alone, and repeat SRT alone between 2012 and 2019 for biopsy-proven brain metastasis recurrence after failure of prior SRT treatment at the site of the target lesion. Inclusion of patients from multiple institutions was performed both for the purposes of increasing the overall cohort counts as well as reducing selection bias given the variation in management schemes at the different centers. The treatment decision process was multifactorial in each patient case with consideration of LITT availability, patient operative candidacy regarding burden of intracranial disease and functional status, and patient preferences. All LITT patients were biopsied at the time of LITT and this study represents the subset of patients with active tumor recurrence, while those with RN were still treated with LITT but not included herein. Post-LITT SRT as either single-fraction radiosurgery (SRS) or fractionated stereotactic radiotherapy (FSRT) was administered per institution practice, typically between 3 and 6 weeks after ablation. Baseline demographic information including sex, age at treatment, primary tumor subtype, Karnofsky Performance Status (KPS), and prior treatment of the target lesion were collected for all patients and compared between cohorts. Radiographic and treatment information including target lesion location, maximum tumor diameter/volume, pretreatment target lesion diameter/volume, and initial/adjuvant SRT dosing parameters was also collected and compared. Our primary outcome was freedom from local progression (FFLP) of the index lesion, defined as the time from treatment to radiographic progression of the treated lesion and symptom development necessitating a change in management, with censorship when nonprogressed at last follow-up or death.

### Radiographic Evaluation

Clinical and radiographic data were analyzed for disease progression or treatment response via a modified Response Assessment in Neuro-Oncology (RANO) criteria. Failure of initial SRT treatment was determined by the treating physician, and diagnosis of tumor recurrence was determined by biopsy for inclusion in the study. After subsequent treatment, local progression was defined as significant radiographic growth >20% of the target contrast-enhanced lesion volume with associated worsening symptomatology that necessitated an escalation in therapy (bevacizumab and/or craniotomy). Sequential 1- × 1-mm volumetric MRIs were obtained before and after treatment and imported into the BrainLab iPlan Cranial 3.0 software for quantitative, semi-automated volumetric analysis of contrast-enhanced lesion volume. Subsequent local progression was not definitively identified as recurrent tumor or RN in all events and was therefore classified as undifferentiated radiographic progression.

### Statistical Analysis

GraphPad Prism version 9 was used for all statistical analyses (GraphPad Software). Categorical and continuous data are described as frequency (percentage) and median (interquartile range [IQR]), respectively. Fisher’s exact test and the Mann–Whitney *U* or Kruskall–Wallis tests were used for the univariate analysis of categorical and continuous data, respectively. Kaplan–Meier method was used to generate clinical outcomes analysis. Multivariate logistic regression analysis was performed in a stepwise manner beginning with all variables of *P* <.2 on univariate analysis iterated to only those of significance to determine the potential independent impact of treatment modality on outcomes, among other variables. A *P* <.05 was considered statistically significant.

## Results

### Cohort Demographics

Retrospective chart review was performed and identified a total of 55 patients who underwent subsequent treatment of biopsy-proven, locally recurrent brain metastasis after initial SRT treatment. Twenty-five patients underwent LITT alone, 21 patients underwent LITT plus adjuvant SRT, and 9 patients underwent repeat SRT alone (Table 1). There were no significant differences in age at treatment, baseline KPS, or primary tumor histology. Groups were similar in their systemic disease status though the LITT cohort trended to more advancement. There was no significant difference in prior chemotherapy or surgery. Prior WBRT was observed more frequently among patients previously treated with SRT alone (*P* = .01) while prior surgery was more common in the LITT-alone cohort (*P* = .05).

**Table 1.** Patient Baseline, Lesion, Treatment (LITT/SRT), and Posttreatment Characteristics

Variable	LITT Alone (*n* = 25, 45%)	LITT+SRT (*n* = 21, 38%)	SRT Alone (*n* = 9, 16%)	*P*-Value
Age at treatment, y (IQR)	56 (47.5–63.5)	60 (57.5–69)	60 (49.5–66)	NS
Male, *n* (%)	7 (28%)	14 (67%)	2 (22%)	**.01**
Baseline KPS, *n* (IQR)	80 (70–90)	90 (80–100)	80 (70–85)	NS
Primary pathology, *n* (%)				NS
NSCLC	15 (60%)	6 (29%)	3 (33%)	
Breast	5 (20%)	4 (19%)	5 (56%)	
Melanoma	1 (4%)	3 (14%)	0 (0%)	
Colon	1 (4%)	3 (14%)	0 (0%)	
Other (renal, esophageal, SCLC)	3 (12%)	5 (24%)	1 (11%)	
Previous surgery	5 (20%)	0 (0%)	1 (11%)	**.05**
Previous WBRT	7 (28%)	0 (0%)	5 (56%)	**.01**
Previous chemotherapy	20 (80%)	15 (71%)	4 (44%)	NS
Laterality (left), *n* (%)	18 (72%)	15 (75%)	4 (44%)	NS
Target lesion location, *n* (%)				NS
Frontal	15 (60%)	5 (25%)	3 (33%)	
Parietal	5 (20%)	8 (58%)	1 (11%)	
Occipital	1 (4%)	1 (8%)	1 (11%)	
Temporal	0 (0%)	1 (0%)	0 (0%)	
Deep	2 (8%)	4 (0%)	1 (11%)	
Cerebellar	2 (8%)	2 (8%)	3 (33%)	
Pre-LITT/biopsy contrast-enhancing volume	5.9 (2.5–8.1)	4.9 (3.3–19.4)	9.3 (3.4–11.5)	NS
LITT characteristics				
Number of trajectories, *n* (range)	1 (1–2)	1 (1–1)	—	NS
Coverage yellow TDT, % (IQR)	98.9% (96.8–100.0)	99.2% (93.0–100.0)	—	NS
Coverage blue TDT, % (IQR)	96.0% (87.8–99.7)	94.3% (81.3–100.0)	—	NS
Hospital length of stay (d), *n* (IQR)	2 (1–3)	1 (1–3)	—	NS
SRT characteristics				
Fractionated SRT, *n* (%)	—	19 (83%)	5 (56%)	NS
SRT total dose, Gray (IQR)	—	25 (25–25)	25 (18–26)	NS
Posttreatment characteristics				
Follow-up, mo (IQR)	5.9 (3.2–9.7)	12.7 (6.8–23.4)	6.2 (5.7–11.3)	**.01**
Radiographic progression, *n* (%)	10 (40%)	5 (24%)	6 (67%)	
Median time, mo	7.5	29.8	3.7	**.02**

Abbreviations: IQR, interquartile range; KPS, Karnofsky Performance Status; LITT, laser interstitial thermal therapy; NS, nonsignificant; NSCLC, non-small cell lung cancer; SCLC, small cell lung carcinoma; SRT, stereotactic radiotherapy; TDT, thermal damage threshold; WBRT, whole brain radiotherapy.

*P* <.05 considered significant and bolded.

### Lesion Radiographic and LITT/SRT Treatment Characteristics

**Figure 1.**
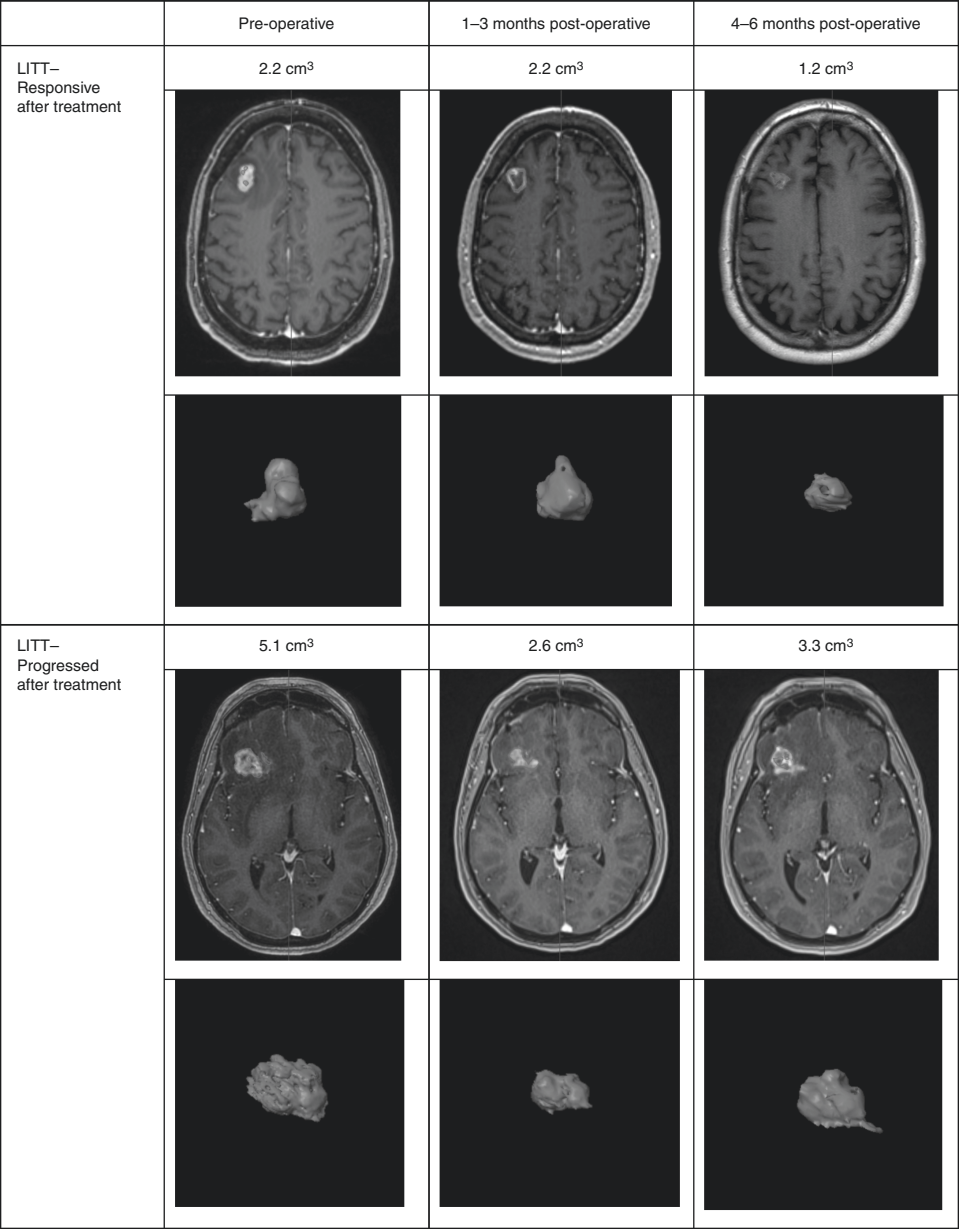
Representative imaging evolution of LITT-treated lesions. The upper images were obtained from T1 post-contrast MRI sequences. The lower images depict volumetric models of the mapped lesions. LITT, laser interstitial thermal therapy.

**Figure 2.**
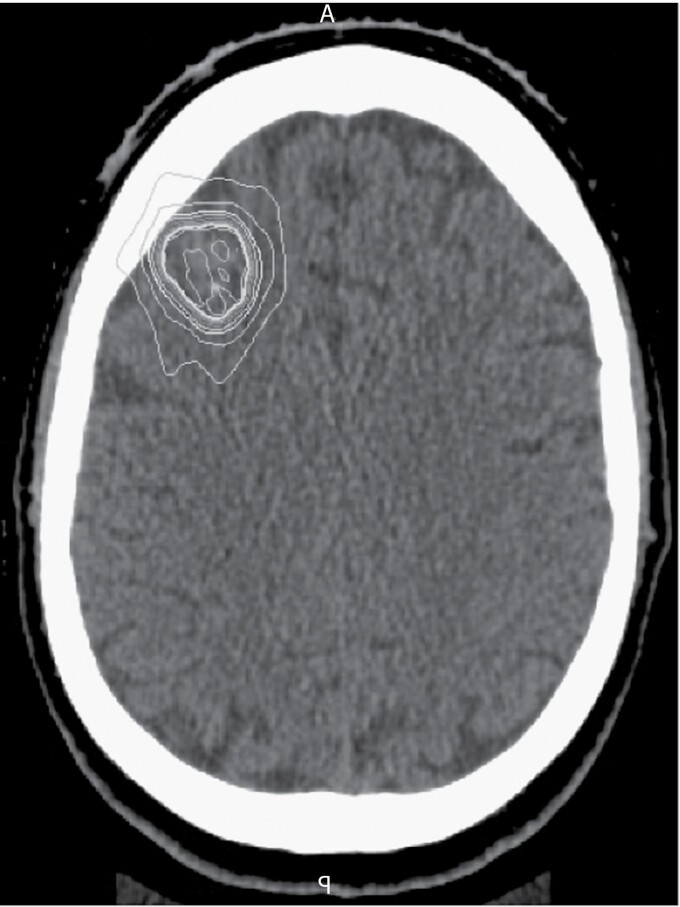
Representative radiation isodose lines for planning post-LITT stereotactic radiotherapy, corresponding to the first case in Figure 1. Obtained from CT scan, the most central line denotes 1890 cGy radiation and the most superficial 540 cGy. LITT, laser interstitial thermal therapy.

The baseline radiographic characteristics of the cohorts are shown in Table 1, with representative imaging in Figures 1 and 2. There were no differences in lesion location or laterality between groups. There was also no statistically significant differences observed in the pre-LITT/biopsy contrast-enhancing volume, though SRT alone trended larger with median volumes of 5.9 cm^3^ (IQR 2.5–8.1), 4.9 cm^3^ (IQR 3.3–19.4), and 9.3 cm^3^ (IQR 3.4–11.5), respectively.

**Figure 3.**
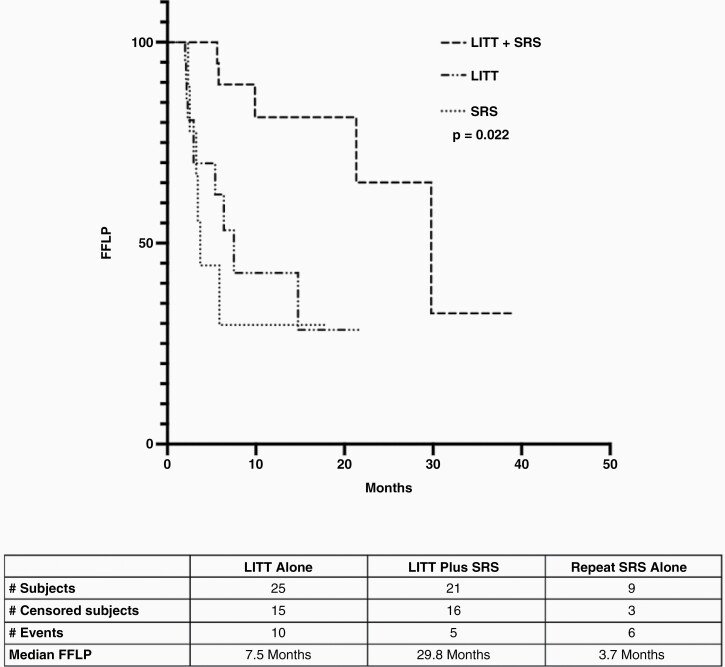
Kaplan–Meier analysis for FFLP by treatment cohort. FFLP, freedom from local progression; LITT, laser interstitial thermal therapy; SRS, single-fraction radiosurgery.

In terms of the treatment characteristics for the SRT-treated cohorts, there was no significant difference between the proportion of patients treated with FSRT or median prescription dose between the LITT+SRT or SRT-alone cohorts (83% vs. 56% fractionated and 25 [IQR 20–30] vs. 25 [IQR 18–26] Gy prescription dose, respectively). For the LITT-treated cohorts, there was no significant difference between the number of trajectories taken, percent coverage to yellow (reversible heat damage) thermal damage threshold (TDT) line, or percent coverage to blue (irreversible heat damage) TDT line between the LITT alone and LITT+SRT groups (1 vs. 1 trajectory, 98.9% vs. 99.2% for the yellow TDT, and 96.0% vs. 94.3% for the blue TDT, respectively). There was also no significant difference in the hospital length of stay (median for LITT alone was 2 days [IQR 1–3] vs. LITT+SRT 1 days [IQR 1–3]).

### Clinical Outcomes by Treatment Cohort

Clinical outcomes for separate treatment cohorts are shown in Table 1. LITT+SRT was associated with lower rates of radiographic local progression (24% compared to 40% for LITT alone and 67% for SRT alone). LITT+SRT was also associated with improved median FFLP at 29.8 months compared to 7.5 and 3.7 months, respectively, with nonprogressed patients censored at last follow-up or death (Figure 2, *P* = .02). In the Kaplan–Meier survival analysis shown in Supplementary Figure 1, LITT+SRT was also associated with improved median OS at 12.7 months compared to 5.9 and 6.2 months for LITT and SRS alone, respectively (*P* = .03).

### Factors Associated With Tumor Progression

On univariate analysis, factors that were significantly associated with decreased likelihood of tumor progression were receiving LITT+SRT, age, and prior surgery. Primary tumor histology (non-small cell lung cancer [NSCLC] vs. others) and pre-LITT/biopsy contrast-enhancing tumor volume did not predict lesion progression (Table 2). Based on the univariate analysis, the following variables met inclusion criteria for the multivariate analysis: treatment modality, age, prior surgery, KPS, and prior WBRT. In a stepwise regression model, the following variables were found to be independent predictors of radiographic progression in our cohorts: treatment modality and age. Patients treated with LITT followed by adjuvant SRT were 5.5 times less likely to have progression of their index lesion, when controlling for age (Table 2, *P* = .004).

**Table 2.** Univariate Analysis of Predictors of Tumor Progression

Variable	Hazard Ratio (95% CI)	*P*-Value
Univariate analysis		
Received LITT vs. repeat SRT	0.51 (0.12–1.60)	.30
Received LITT+SRT vs. others	0.45 (0.21–0.92)	**.03**
Age	0.94 (0.91–0.98)	**.001**
Sex, male	0.79 (0.39–1.62)	.52
Baseline KPS	0.97 (0.95–1.00)	.10
Primary tumor histology (NSCLC/other)	1.02 (0.50–2.04)	.94
Prior treatment		
WBRT	2.02 (0.79–4.52)	.11
Surgery	5.33 (1.41–16.93)	**.007**
Pre-LITT/biopsy contrast-enhancing volume (cc)	1.04 (0.95–1.12)	.36
Multivariate analysis		
Age	0.95 (0.91–1.0)	**.04**
Treatment modality (LITT+SRT)	0.18 (0.05–0.53)	**.004**

Abbreviations: KPS, Karnofsky Performance Status; LITT, laser interstitial thermal therapy; NSCLC, non-small cell lung cancer; SRT, stereotactic radiotherapy; WBRT, whole brain radiotherapy.

*P* <.05 considered significant and bolded.

## Discussion

To our knowledge, this is the first multi-institutional study to investigate the relative efficacy of combining LITT with SRT for patients with recurrent brain metastases following SRT failure. In this unique cohort of patients with all biopsy-proven recurrent tumor, outcomes were compared to patients treated with LITT alone or SRT alone. We observed that combining LITT with SRT significantly improves time to index lesion progression, represented by FFLP, after a median follow-up of 7.3 months.

The synergistic effect of combining SRT with LITT is hypothesized to stem from various biologic mechanisms. Prior studies suggest that hyperthermia (HT) may enhance the efficacy of tumor-directed treatment through: (1) its cytotoxic/cytoreductive effect on tumor, independent of oxygenation or cell-cycle status; (2) enhancement of radiosensitivity via re-oxygenation, DNA damage, and inhibition of both lethal and sublethal damage repair; (3) improvement in drug delivery, uptake, and sensitivity; and (4) activation of host antitumor immune responses.^33–38^ Several phase III trials report improved local control by combining HT with radiation in patients with melanoma, breast, head and neck, and esophageal cancer.^7,12,13,39,40^ Additionally, studies suggest that HT does not increase the incidence or severity of normal tissue complications from radiation. However, adoption of HT has historically been limited due to the invasive nature of thermometry and challenges for accurate delineation and calculation of thermal doses. MRI-guided LITT now offers a minimally invasive solution to leverage the antitumor effects of HT and its potential synergy with radiation to improve patient outcomes.

The use of MRI-guided LITT in conjunction with postoperative SRT for patients with recurrent tumor after SRT failure is an active area of investigation. The case series of 20 patients treated with LITT and SRT reported by Peña Pino et al. demonstrated 100% local control rates at 6 and 12 months.^41^ While SRT is a widespread first-line treatment for the management of single and multiple brain metastases, recurrent tumor and RN occur in 5%–15% of SRT-treated brain lesions.^42,43^ As OS continues to improve with the advent of more effective systemic therapies, the management of recurrent disease presents a growing neuro-oncologic challenge. LITT is currently indicated for the treatment of primary and metastatic brain tumors, as well as RN and epilepsy.^12,18,29^ The ability to reliably obtain an accurate pathological diagnosis via concomitant biopsy and deliver effective ablative therapy represents a technological advance. Specifically, improvements in MRI-guided thermometry and the availability of intraoperative MRI now enable surgeons to accurately monitor heat delivery to target tissues and to stereotactically position the laser fiber within the tumor bed. LITT has an excellent safety profile, with multiple studies reporting short hospitalizations and low complications rates.^26,27,29,31^ Work by Hong et al. additionally suggests that LITT is an effective treatment modality for the management of either recurrent tumor or RN following SRT failure. In a cohort of 75 patients, they observed equivalent progression-free survival (PFS), neurologic symptoms, and ability to taper off steroids when compared to craniotomy.^30^ Our study is the first investigation that directly compares the efficacy of combined modality treatment to LITT or SRT alone in the recurrent setting.

The multi-institutional nature of this study, strict inclusion criteria regarding biopsy-proven recurrence, and the use of a single guiding principle for treatment decisions strengthen confidence in these results. Local progression was determined using both RANO-BM criteria and a change in symptomatology necessitating further treatment. In this study, the median FFLP for patients treated with LITT plus SRT was 29.8 months. This compares favorably to the LITT-alone cohort, in which prior WBRT was more common, and for which the median FFLP was 7.5 months. These outcomes agree with findings from Hong et al. and Rao et al., who reported a 6-month PFS of 75.6% and 75.8%, respectively, with LITT alone after SRT failure.^30,31^ The difference in prior WBRT between the cohorts is largely a function of the SRT-alone cohort being treated at earlier timepoints when WBRT was a more widely used treatment modality. This could also reflect differences in prognostic category, though that is less likely to influence subsequent local progression than it would OS measures. Although the difference was not statistically significant, there was higher percentage of fractionation in the LITT+SRT group (83% in LITT+SRT group, 56% in SRT group), which may have been a result of expected lesion expansion post-LITT. A hypothesized rationale for a combinatorial approach emerges in which LITT treats both recurrent tumor and any component of RN within the lesion, and the addition of FSRT eliminates any tumor at the periphery that was left viable despite LITT.

Approximately 50% of tumor histologies were either NSCLC or melanoma, and the impact of targeted therapy and immunotherapy on overall outcomes on this patient population is well documented. Further characterization of tumor heterogeneity and molecular classifications would be beneficial given the demonstrated impact on outcomes; however, these data were not readily available for all patients in the present study.^44,45^ There was no difference in age, histology, performance status, or pretreatment lesion volume between cohorts. The improvement in FFLP among patients treated with combined LITT and SRT may be due to changes in the local and systemic tumor microenvironment induced by HT. Enhancement of drug delivery and sensitivity in the setting of concurrent systemic therapy or activation of a more robust antitumor immune response may play an important role.^46^ Importantly, OS in these patients is a polyfactorial outcome not solely dependent on intracranial disease and, as such, FFLP may be the more relevant metric for consideration. Our analysis did indicate a potential advantage in OS for the LITT+SRT cohort; however, this metric is more susceptible to a range of confounders including volume, additional intracranial lesions, tumor genetics, and systemic disease status, and so should be considered with caution. Future research is necessary to elucidate the impact of LITT+SRT on the tumor microenvironment and whether combined modality treatment yields improved survival.

Despite our study’s robust findings, it is limited by its retrospective nature, small sample size, and limited follow-up time. Additionally, the observed outcomes in the SRT-alone cohort were worse than that previously described in the literature. In our analysis, patients retreated with SRT alone had worse outcomes, with a median FFLP of 3.7 months. This is at odds with prior work by Kurtz et al., in which the reported 6-month local control rate was 83% following SRT for previously irradiated tumors.^16^ This discrepancy is likely due to limited sample size in this investigation and stringent patient selection. Importantly, patients included in our study required biopsy confirmation of recurrent tumor. In contrast, many other studies define recurrent disease radiographically, without mandating biopsy confirmation, thus suggesting that differences in outcomes may stem from misclassification of RN as recurrent tumor. This pitfall is one that our study was designed specifically to avoid. Additionally, the intracohort differences between median follow-up and FFLP are due to censoring with patient deaths from systemic causes prior to intracranial progression or loss to follow-up. Larger sample sizes would better control for variability, though the median follow-up for LITT plus SRT is still superior relative to the FFLP in the LITT- or SRT-alone cohorts. Finally, selection of patients for LITT+SRT, LITT alone, or SRT alone was at the discretion of the treating provider. While there were no differences in baseline characteristics between cohorts, the SRT-alone cohort trended towards larger lesions and it is unclear whether tumor histology or other confounding variables influencing treatment selection (bias of indication) may have contributed to the observed differences in outcomes.

In conclusion, the results of this study suggest that combination LITT+SRT may represent a more effective alternative to LITT alone or SRT alone in patients with biopsy-proven recurrent metastatic intracranial tumor following SRT failure. With comparable retrospective study cohorts, we found that LITT+SRT led to significantly prolonged FFLP. The low complication rate with combined modality therapy (despite similarities in radiotherapy fractionation rates among groups) suggests that this novel treatment strategy is both efficacious and safe. This treatment may be particularly effective for patients with poor performance status or deep-seated lesions for whom surgical intervention could compromise neurologic function. Future prospective studies are needed to validate these results and to determine whether LITT+SRT improves FFLP and other clinical outcome measures.

## Supplementary Material

vdac086_suppl_Supplementary_Figure_S1

## Notes

**Authorship Statement**. Experimental design: M.M.G., E.S.S., E.W.S., and P.E.F.; implementation: M.M.G., E.S.S., E.J.V., E.W.S., B.O., D.K., A.S., M.O., J.M., and E.I.F.; analysis and interpretation: all authors.
