# Supplementary figures and images for: Combination laser interstitial thermal therapy plus stereotactic radiotherapy increases time to progression for biopsy-proven recurrent brain metastases
Source: Neurooncol Adv. 2022 Jun 2;4(1):vdac086. doi: 10.1093/noajnl/vdac086 (PMC9248774; doi:10.1093/noajnl/vdac086)

Supplemental Figure 1: Kaplan-Meier analysis for overall survival (OS) by treatment cohort.


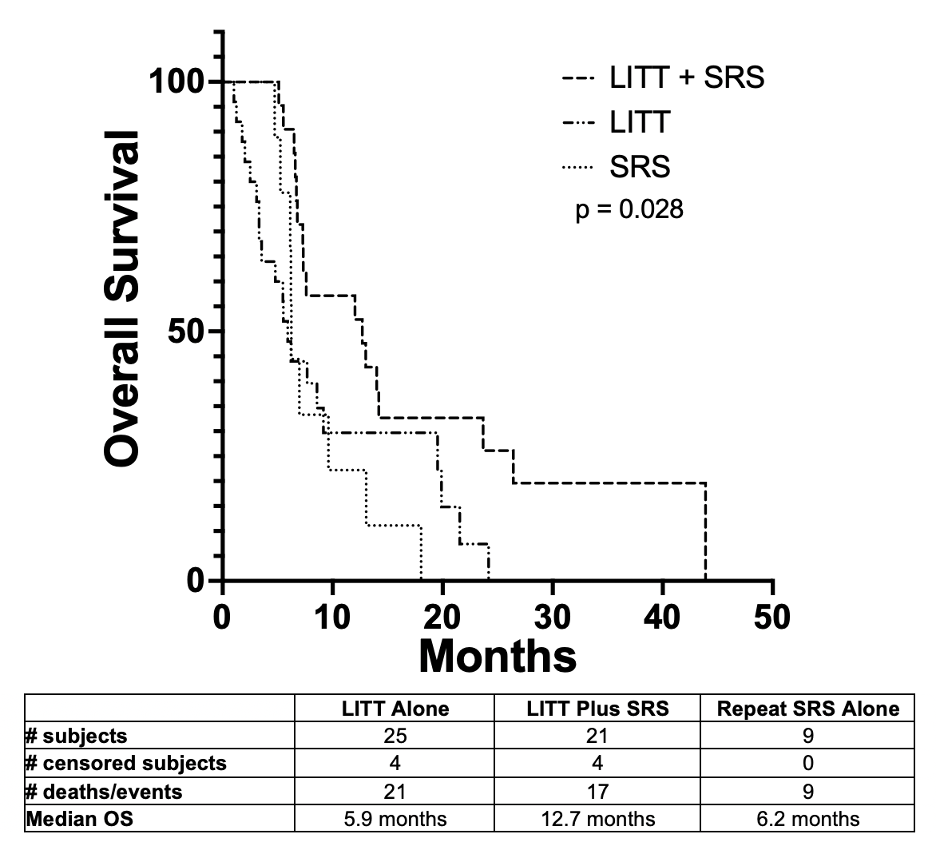

Supplement: vdac086_suppl_Supplementary_Figure_S1 [file vdac086_suppl_supplementary_figure_s1.docx]
